# Sapovirus Gastroenteritis in Preschool Center, Puerto Rico, 2011

**DOI:** 10.3201/eid1901.120690

**Published:** 2013-01

**Authors:** Encijar Hassan-Ríos, Pilar Torres, Elizabeth Muñoz, Carmen Matos, Aron J. Hall, Nicole Gregoricus, Jan Vinjé

**Affiliations:** Author affiliations: Puerto Rico Department of Health, San Juan, Puerto Rico (E. Hassan-Ríos, P. Torres, E. Muñoz, C. Matos);; Centers for Disease Control and Prevention, Atlanta, Georgia, USA (A.J. Hall, N. Gregoricus, J. Vinjé)

**Keywords:** sapovirus, viruses, gastroenteritis, outbreak, preschool center, Puerto Rico

**To the Editor:** Human sapoviruses belong to a group of viruses within the family *Caliciviridae*, which also includes noroviruses, that cause acute gastroenteritis (*1*). Evidence of worldwide distribution of sapovirus has been documented on the basis of detection of virus and antibody prevalence against sapovirus in different populations (*2*). However, no evidence of sapovirus infection or outbreaks in Latin America and the Caribbean Islands has been reported.

In this study, we describe a sapovirus-associated outbreak of gastroenteritis in a preschool center during February–March 2011 in Canóvanas, Puerto Rico. The center had 60 children 4–5 years of age enrolled who were divided in 3 groups of 20 students per classroom. Each classroom had 2 teachers. The children had lunch in their respective classrooms.

A study was conducted at the center and included all children and workers who met the case definition for gastroenteritis (vomiting or diarrhea accompanied by >1 other symptom, such as nausea, stomach ache, or fever, during February 15–March 15, 2011). Nine persons (8 students and 1 teacher) met the case definition. They were interviewed by using a standardized questionnaire. Major symptoms were vomiting (100%), nausea (71.4%), fever (62.5%), stomach ache (57.1%), and diarrhea (25%) (Table). Data for fever were based on subjective reports of case-patients.

**Table:** Clinical symptoms and laboratory results for 9 patients with acute gastroenteritis in preschool center, Puerto Rico, 2011*

Patient	Age, y/sex	Date of illness onset	Date of specimen collection	Norovirus RT-PCR result	Sapovirus RT-PCR result	Vomiting		Nausea		Fever		Stomach ache	Diarrhea	
1	5/M	Feb 23	NS	NS	NS	+		+		–		–	–	
2	5/F	Mar 2	NS	NS	NS	+		U		+		U	–	
3	4/F	Mar 2	Mar 11	–	+	U		U		+		U	+	
4	5/F	Mar 3	Mar 14	–	+	+		+		+		–	–	
5	5/F	Mar 3	Mar 14	–	+	+		+		+		–	–	
6	4/F	Mar 3	NS	NS	NS	+		–		+		+	–	
7	4/F	Mar 7	Mar 9	NT	NT	+	–		+		+			–
8	53/F	Mar 8	Mar 11	NT	NT	+	+		–		+			+
9	4/M	Mar 8	Mar 9	NT	NT	U	+		–		+			+

*RT-PCR, reverse transcription PCR; NS, no sample; +, positive; –, negative; U, unknown (missing information); NT, not tested.

The earliest date of illness onset identified in the outbreak was February 23 in a child whose symptoms began abruptly with a vomiting event in the classroom. The child had a second vomiting event in the bathroom before the child was sent home. An initial cleaning was made with an absorbent powder, and a chlorine bleach solution was used for disinfection. That child was absent from school on February 24 and 25 (Thursday and Friday) and returned to school on Monday, February 28, supposedly recovered. The next reported illnesses began on March 2.

Fecal specimens were collected from 6 ill persons who met the case definition. The specimens were collected 2–11 days after onset of illness. All specimens were negative for enteric bacteria. Three specimens were sent to the Centers for Disease Control and Prevention (Atlanta, GA, USA) for virologic analysis. All 3 specimens were negative for norovirus and positive for sapovirus by real-time quantitative reverse transcription PCR (Table).

An environmental inspection and evaluation was conducted at the preschool center and showed no deficiencies. Neither of the 2 food handlers associated with the school reported symptoms of gastroenteritis. Fecal specimens collected from both food handlers were negative for enteric bacteria but were not tested for viral pathogens. Sapovirus transmission from asymptomatic food handlers in foodborne outbreaks has been reported (*3*). However, sapoviruses are much less frequently associated with foodborne outbreaks than are noroviruses (*4*).

Transmission during this outbreak most likely occurred person-to-person directly through fecal–oral contact or by indirect exposure through contaminated objects or surfaces because 7 (78%) of the 9 ill persons were from the same classroom (attack rate 32% [7 of 22 students and teachers]). The other 2 ill persons were a child in a different classroom who was a cousin of 1 of the ill children in the affected classroom and a teacher from the other classroom that shared the bathroom with the affected classroom.

This investigation highlights the need for clinical diagnostics of viral pathogens in evaluation of persons with acute gastroenteritis. A recent study in the United States demonstrated that viruses were the leading cause of acute gastroenteritis among persons of all ages seeking medical care (*5*). Better understanding of the relative role of specific causes of acute gastroenteritis is needed to help guide clinical management and ultimately to develop more appropriate prevention strategies. Limited laboratory-based data are available on the role of viral agents in causing acute gastroenteritis for sporadic cases and outbreaks in Puerto Rico. On the basis of this investigation, sapoviruses appear to be circulating in Puerto Rico and should be considered a potential cause of gastroenteritis in children and adults. We recommend expanded use of sapovirus diagnostics in other Latin American countries and Caribbean Islands to better elucidate their role in cases of viral gastroenteritis.
